# Comparison of endoscopic ultrasound-guided drainage and percutaneous drainage combined with minocycline sclerotherapy for symptomatic hepatic cysts: A retrospective study

**DOI:** 10.1097/MD.0000000000037677

**Published:** 2024-03-29

**Authors:** Taiji Yoshimoto, Takeshi Takajo, Hirokazu Iijima, Ryuichi Yamamoto, Hiroshi Takihara, Fumiya Nishimoto

**Affiliations:** a Department of Gastroenterology, Musashino Tokushukai Hospital, Tokyo, Japan; b Department of Surgery, Musashino Tokushukai Hospital, Tokyo, Japan; c Department of Gastroenterology, Tokyo-west Tokushukai Hospital, Tokyo, Japan; d Department of Gastroenterology, Uji Tokushukai Hospital, Uji, Kyoto, Japan; e Division of Gastroenterology Department of Internal Medicine Showa University Fujigaoka Hospital Kanagawa Japan.

**Keywords:** EUS-guided drainage, symptomatic hepatic cysts, percutaneous drainage, sclerotherapy, minocycline

## Abstract

Simple hepatic cysts (SHC) are generally asymptomatic and incidentally diagnosed using imaging studies. Asymptomatic SHC does not require treatment, but symptomatic SHC warrants treatment using different modalities, including intravenous antibiotic therapy, ultrasound-guided percutaneous catheter drainage (PCD) with sclerotherapy, and surgery.

The dissemination of endoscopic ultrasonography (EUS) intervention techniques has enabled the performance of puncture and drainage via the transgastrointestinal route for intra-abdominal abscesses. Despite the development of an EUS-guided drainage method for treating symptomatic SHC, only a few case reports using this method have been reported.

This study retrospectively analyzed the safety and feasibility of EUS-guided drainage of symptomatic SHC as well as its clinical outcomes and compared it with combined therapy using PCD and minocycline sclerotherapy. The records of 10 consecutive patients with 11 symptomatic SHCs treated with either EUS-guided drainage or PCD combined with minocycline sclerotherapy at the Musashino Tokushukai Hospital from August 2019 to January 2024 were retrospectively examined.

All cases in both groups achieved technical and clinical success, with no reported adverse events. The median reduction rates of the major cyst diameters in the EUS-guided drainage and PCD with sclerotherapy groups were 100% (interquartile range [IQR]: 94%–100%) and 67% (IQR: 48.5%–85%). The length of hospital stay was 7 and 22.5 days in the EUS-guided and PCD with sclerotherapy groups (*P = *.01).

EUS-guided drainage of symptomatic SHC is a safe and effective therapeutic alternative to percutaneous drainage with sclerotherapy and surgery for treating symptomatic SHC.

## 1. Introduction

Simple hepatic cysts (SHC) are generally asymptomatic and are diagnosed incidentally using imaging studies. The prevalence of SHC is reported to range from 2.5% to 18.0%.^[1,2]^ It is more commonly found in women and the elderly population as compared with men and young population. Asymptomatic SHC does not require treatment, however symptomatic SHC needs to be treated and can be treated using different treatment modalities such as intravenous antibiotic therapy, ultrasound-guided percutaneous catheter drainage (PCD) with sclerotherapy, and surgery.^[3–9]^ Following publication of the first report on endoscopic ultrasonography (EUS)-guided drainage of pancreatic pseudocysts, this technique has been widely applied in several fields^[10–13]^; however, case reports describing EUS-guided drainage of symptomatic SHC are scarce.^[14–18]^ In addition, there has been no comparison study of EUS-guided drainage and PCD combined with minocycline sclerotherapy.

Therefore, the aim of this study was a retrospective analysis of the safety and feasibility of EUS-guided drainage of symptomatic SHC and the clinical outcomes of EUS-guided drainage while comparing it with the combined therapy of PCD and minocycline sclerotherapy.

## 2. Methods

The records of 10 consecutive patients with 11 symptomatic SHCs, who were treated with either EUS-guided drainage or PCD combined with minocycline sclerotherapy at Musashino Tokushukai Hospital between August 2019 and January 2024 were retrospectively examined in this study. The characteristics of patients including sex, age, clinical symptoms, and blood test results and the characteristics of the hepatic cysts including cyst location and size, and presence of solitary or multiple cysts, were investigated. The results of each treatment, including the technical and clinical success rate, posttreatment cyst size and reduction rate, hospital stay, follow-up period, total usage of minocycline, recurrence rate, and adverse events, were analyzed. The Institutional Review Board of Musashino Tokushukai Hospital approved this study. Written informed consent was obtained from all participating patients.

### 2.1. Endoscopic ultrasonography-guided drainage

**Figure 1.**
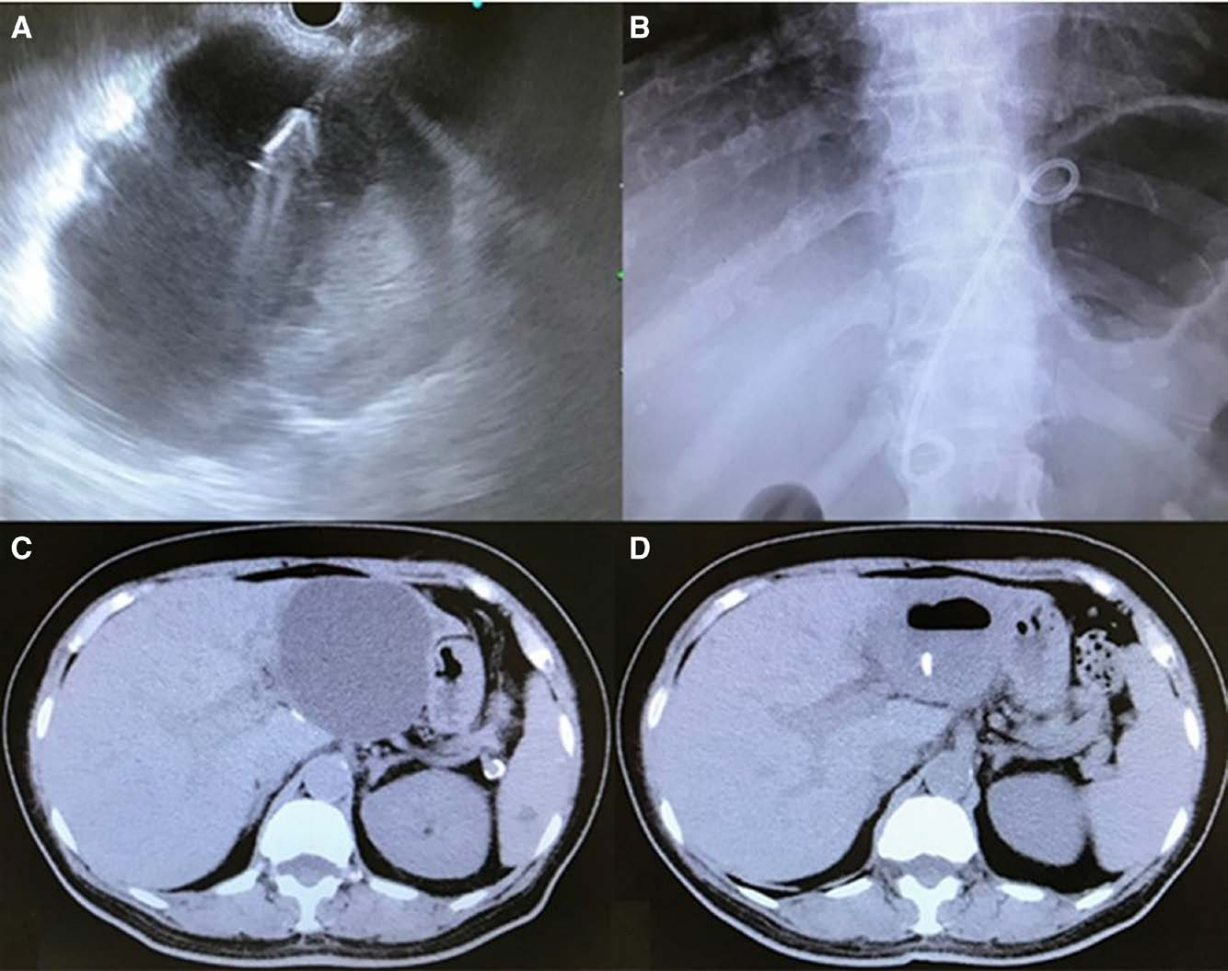
EUS-guided drainage of symptomatic SHC. (A) 19-gauge needle is inserted into the cavity. (B) A 7-Fr 10 cm DPPS is placed. (C) CT scan reveals a large cyst in left lobe of the liver. (D) CT scan confirms that the drainage tube is placed in the cavity of the hepatic cyst. CT = computed tomography, DPPS = double pigtail plastic stent, SHC = simple hepatic cysts.

A convex array echoendoscope (GF-UCT260; Olympus Medical Systems, Tokyo, Japan) and EU-ME2 PREMIER PLUS (Olympus Medical Systems) were used for performing EUS-guided transgastric drainage with intravenous flunitrazepam (Midazolam^®^; Nichi-Iko Pharmaceutical Co., Ltd., Toyama, Japan) and pethidine hydrochloride (pethidine hydrochloride^®^; Takeda Pharmaceutical Company Limited., Tokyo, Japan) under sedation. The cyst fluid was aspirated by puncturing the hepatic cyst with a 19-gauge fine needle aspiration (fine needle aspiration) needle (EZ Shot 3 Plus; Olympus Medical Systems) under color Doppler visualization to avoid puncturing any intervening vessels. A contrast medium was injected to confirm the cystic cavity followed by the insertion of a 0.025-inch guidewire (EndoSelector; Boston Scientific, Massachusetts, United States) through the fine needle aspiration needle. After the guidewire was coiled within the cyst cavity, a 7-Fr double pigtail plastic stent (DPPS; Through & Pass^TM^ Stent; Gadelius Medical Co, Ltd., Tokyo, Japan) or straight plastic stent (Straight Plastic Stent; Flexima; Boston Scientific, Natick, Mass, USA) was deployed within the cystic cavity. The correct placement of the drainage tube in the cyst cavity was confirmed by a CT after the procedure (Fig. 1).

### 2.2. Percutaneous catheter drainage using minocycline sclerotherapy

A Toshiba Aplio 400 device (Toshiba Medical Systems Corporation, Otawara, Japan) was used to perform ultrasound-guided PCD with intravenous flunitrazepam (Midazolam^®^; Nichi-Iko Pharmaceutical Co., Ltd.) under sedation. The puncture site was anesthetized with 1% lidocaine hydrochloride monohydrate (Xylocaine^®^; Sandoz K.K., Tokyo, Japan) and the hepatic cyst was punctured with an 18-gauge needle (The CLINY PTCD KIT one step type; CREATE MEDIC CO., LTD., Kanagawa, Japan) under ultrasonography guidance for aspiration of the cyst fluid. The next step was the injection of a contrast medium to confirm that the cystic cavity had no communication with the biliary duct or extravasation into the peritoneal cavity. A 0.035-inch guidewire (The CLINY PTCD KIT one step type; CREATE MEDIC CO., LTD.) was introduced through the needle, and after the guidewire had coiled within the cystic cavity, a 7-Fr pigtail long catheter (The CLINY PTCD KIT one step type; CREATE MEDIC CO., LTD.) was deployed within the cystic cavity using a 7-Fr dilator (The CLINY PTCD KIT one step type; CREATE MEDIC CO., LTD.). A CT scan confirmed the placement of the drainage tube in the cyst cavity.

### 2.3. Protocol of minocycline sclerotherapy

**Figure 2.**
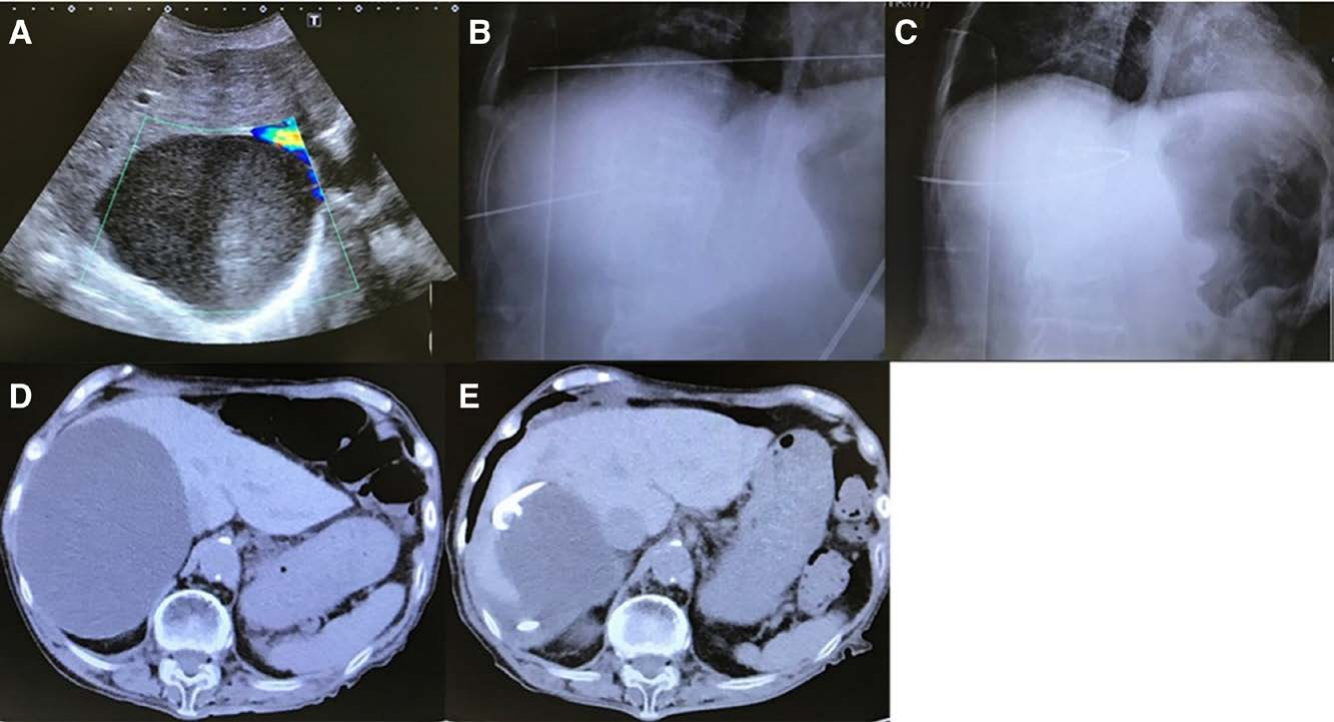
PCD with minocycline sclerotherapy for treating symptomatic SHC. (A) Ultrasound indicates a large cyst in right lobe of the liver. (B) An 18-gauge needle is advanced into the cavity. (C) A 7-Fr pigtail long catheter is placed. (D) CT scan reveals a large cyst in right lobe of the liver. (E) CT scan confirms that the drainage tube is placed in the cavity of the hepatic cyst. CT = computed tomography, PCD = percutaneous catheter drainage, SHC = simple hepatic cysts.

Minocycline hydrochloride (100 mg) (MINOCYCLINE HYDROCHLORIDE^®^; Sawai Pharmaceutical Co., Ltd., Osaka, Japan) dissolved in 5 mL of normal saline and 5 mL of 1% lidocaine hydrochloride monohydrate (Xylocaine^®^; Sandoz K.K.) (10 mL in total) was injected after completely aspirating the cyst fluid. The catheter was clamped for half a day (12 h) after flushing it with 5 mL of normal saline. After confirming the absence of side effects of minocycline, based on continued drainage (>20–30 mL/d), minocycline at 100 mg was continued or increased to 200 mg. Minocycline sclerotherapy was administered daily, and the catheter was removed when no reaccumulation was observed (Fig. 2).

### 2.4. Statistical analysis

Categorical variables were compared using Fisher’s exact test and normally or nonnormally distributed continuous variables were compared using Student *t* test or Man–Whitney *U* test. *P* < .05 was considered statistically significant. All statistical analyses were performed using SPSS version 29.0 for windows (IBM Japan Ltd., Tokyo, Japan).

## 3. Results

### 3.1. Participants and descriptive data

Total 10 patients with 11 symptomatic SHCs were enrolled in this study, among which 4 patients (2 males and 2 females) with 4 hepatic cysts were treated with EUS-guided drainage (EUS-guided drainage group), and 6 patients (1 male and 5 females) with 7 hepatic cysts were treated with PCD along with simultaneous minocycline sclerotherapy (PCD with minocycline sclerotherapy group). No significant differences were noted in patient and hepatic cyst characteristics between both groups, except for cyst location (Table 1).

**Table 1:** Characteristics of patients and hepatic cysts.

	EUS-guided drainage n = 4	PCD with minocycline Sclerotherapy n = 6	*P*-value
Age, yr (IQR)	67.5 (66–71.2)	83.5 (79–85.8)	.17
Male/female, n (%)	2/2 (50/50)	1/5 (16/74)	.26
Location			<.001
Left lobe	4	0	
Right lobe	0	7	
Solitary/multiple	1/3	0/6	.40
Major cyst diameter, mm (IQR)	71 (44–109.2)	124 (100.5–140.5)	.18
Clinical symptoms (fever, abdominal pain)	4	5	.39
Blood tests			
Elevated Inflammation markers (WBC, CRP)	4	4	.20
Elevated liver enzymes (AST, ALT, γ-GTP) or Bil	4	6	1.0

ALT = alanine aminotransferase, AST = aspartate aminotransferase, Bil = bilirubin, CRP = C-reactive protein, IQR = interquartile range, PCD = percutaneous catheter drainage, WBC = white blood cell; γ-GTP = γ-glutamyl transpeptidase.

### 3.2. Outcomes

Technical and clinical success was achieved in all cases in both groups, and no adverse events were reported. 100% (interquartile range [IQR], 94%–100%) and 67% (IQR, 48.5%–85%) were the median reduction rates of the major cyst diameters in the EUS-guided drainage and the PCD with sclerotherapy groups, respectively. The median total usage of minocycline per cyst was 1000 mg (IQR: 500–1150 mg) and the median number of sclerotherapy sessions was 5 (IQR: 5–9 sessions). The follow-up period was 21 months for the EUS-guided drainage and 33.9 months for the PCD with sclerotherapy groups. The length of hospital stay was 7 and 22.5 days in the EUS-guided group and PCD with sclerotherapy group, respectively (*P = *.01, retrospectively). Neither groups reported any recurrence (Table 2).

**Table 2:** Clinical outcomes.

	EUS-guided drainage n = 4	PCD with minocycline sclerotherapy n = 6	*P*-value
Technical success, n (%)	4 (100)	6 (100)	
Clinical success, n (%)	4 (100)	6 (100)	
Adverse events, n (%)	0 (0)	0 (0)	
Major cyst diameter after treatment, mm (IQR)	0 (0–9.25)	41 (18.5–63.5)	.11
Reduction rate of major cyst diameter, % (IQR)	100 (94–100)	67 (48.5–85)	.11
The length of hospital stay, days (IQR)	7.5 (5.5–9)	16 (14–19.5)	.01
Follow-up period, months (IQR)	18 (4.8–34.2)	41 (17.1–48.8)	.34
Recurrence rate, %	0	0	
Total usage of minocycline per cyst, mg (IQR)	0	1000 (500–1150)	
Sclerotherapy sessions, times (IQR)	0	5 (5–9)	
Stent removal	3 cases (3.5, 6, and 20 mo later)	All cases before discharge	
Stent type	7-Fr (DPPS 3 and SPS 1)	All cases 7-Fr pigtail catheter	

DPPS = double pigtail plastic stent, IQR = interquartile range, PCD = percutaneous catheter drainage, SPS = straight plastic stent.

## 4. Discussion

The dissemination of EUS intervention techniques has enabled performing puncture and drainage via the transgastrointestinal route for intra-abdominal abscesses. Despite development of EUS-guided drainage method for treating symptomatic SHC, only a few case reports of this method have been reported. The aims of the current study were retrospective investigation of the feasibility and safety of EUS-guided drainage of symptomatic SHC and comparison of the clinical outcomes of this drainage method with the combined treatment of PCD and minocycline sclerotherapy. The results of our retrospective study indicate achievement of technical and clinical success in all cases without adverse events. The length of hospital stay was significantly shorter in the EUS-guided drainage group compared with the PCD combined with minocycline sclerotherapy group (7 d vs 22.5 d, *P = *.01, retrospectively).

The advantages of EUS-guided drainage are as follows: Clear visualization of the hepatic cyst without skin and rib artifacts; Color Doppler mode enables performing punctures while avoiding blood vessels; the absence of pain due to percutaneous punctures; and Absence of the risk of self-tube removal and side effects due to sclerosing agents.

No recurrence was observed in the EUS-guided drainage group during the follow-up period of 18 months. This indicates that the destruction of epithelial cells that secrete fluid within the hepatic cyst using a sclerosing agent is not always necessary. Plastic stents can function as foreign bodies that promote granulation, covering the epithelial cells lining the cyst cavity, and preventing fluid secretion.

After complete disappearance of hepatic cysts, the internal plastic stents were removed in 3 cases (3.5, 6, and 20 mo later, retrospectively), and no recurrence was observed after stent removal. Recently, D’Errico et al reported EUS-guided drainage using lumen-apposing metal stents for treating symptomatic SHC safely and with high clinical success rates.^[19]^ They exchanged the lumen-apposing metal stents with a DPPS 1 month later and then removed the DPPS 3 months later. Although the need to remove internal stents and the appropriate time of stent removal are controversial, a fistula generally forms in approximately 1 month, and making stent removal feasible at that time.

A systematic review conducted by Furumaya et al reported regarding treating symptomatic SHC using PCD with sclerosing agents, such as ethanol or minocycline, versus surgery, has proposed a step-up treatment strategy in which the primary treatment is PCD with sclerotherapy, the second-line treatment is laparoscopic cyst deroofing, and the final-line treatment is open hepatic resection.^[20]^ Shionoya et al reported a case in which symptomatic SHC with biliary communication was treated using EUS-guided drainage. In such cases, PCD with sclerotherapy is often ineffective, and EUS-guided drainage may be the first choice.^[21]^ In our opinion, EUS-guided drainage can potentially be used as a first-line treatment for symptomatic SHC patients in facilities with skilled endoscopists.

As the number of elderly patients is on the rise worldwide, less invasive and more effective methods are required for symptomatic SHC drainage. Few previously conducted studies have reported that the technical and clinical success rates of EUS-guided drainage for hepatic abscesses were 88.9% to 100% and 71.4% to 100%, and the recurrence rates were 0% to 7%, retrospectively.^[22–24]^ Although a prospective study involving a larger number of patients is required to confirm the utility of EUS-guided drainage for symptomatic SHC, this seems to be an emerging method with high technical and clinical success rates similar to percutaneous drainage combined with sclerotherapy. Moreover, this method may shorten the length of hospital stay.

Limitations of the current study include the following. First, the EUS-guided drainage was not compared with PCD using other sclerosants, such as ethanol. Second, this study did not attempt to perform EUS-guided drainage for the right hepatic cysts. Third, a single endoscopist performed all EUS-guided drainages. Finally, this was a retrospective single-center study with a small sample size.

In conclusion, EUS-guided drainage of symptomatic SHC is a safe and effective therapeutic alternative to percutaneous drainage with sclerotherapy and surgery for treating symptomatic SHC; however, large-scale investigations are required to confirm the present findings.

## Author contributions

**Conceptualization:** Taiji Yoshimoto, Takeshi Takajo, Hirokazu Iijima, Ryuichi Yamamoto, Hiroshi Takihara, Fumiya Nishimoto.

**Data curation:** Taiji Yoshimoto.

**Formal analysis:** Taiji Yoshimoto, Hiroshi Takihara.

**Investigation:** Taiji Yoshimoto, Takeshi Takajo, Hirokazu Iijima.

**Methodology:** Taiji Yoshimoto.

**Project administration:** Taiji Yoshimoto, Takeshi Takajo, Hirokazu Iijima, Hiroshi Takihara.

**Resources:** Taiji Yoshimoto, Takeshi Takajo, Hirokazu Iijima, Fumiya Nishimoto.

**Software:** Taiji Yoshimoto.

**Supervision:** Taiji Yoshimoto.

**Validation:** Taiji Yoshimoto, Hiroshi Takihara.

**Visualization:** Taiji Yoshimoto.

**Writing – original draft:** Taiji Yoshimoto.

**Writing – review & editing:** Taiji Yoshimoto, Hiroshi Takihara.
